# Promising and challenging phytochemicals targeting LC3 mediated autophagy signaling in cancer therapy

**DOI:** 10.1002/iid3.70041

**Published:** 2024-10-22

**Authors:** Peramaiyan Rajendran, Kaviyarasi Renu, Enas M. Ali, Marwa Azmy M. Genena, Vishnupriya Veeraraghavan, Ramya Sekar, Ashok Kumar Sekar, Sujatha Tejavat, Parthasarathi Barik, Basem M. Abdallah

**Affiliations:** ^1^ Department of Biological Sciences, College of Science King Faisal University Al‐Ahsa Saudi Arabia; ^2^ Department of Biochemistry, Centre of Molecular Medicine and Diagnostics (COMManD), Saveetha Dental College & Hospitals, Saveetha Institute of Medical and Technical Sciences Saveetha University Chennai Tamil Nadu India; ^3^ Department of Botany and Microbiology, Faculty of Science Cairo University Cairo Egypt; ^4^ Agricultural Zoology Department, Faculty of Agriculture Mansoura University Mansoura Egypt; ^5^ Department of Oral & Maxillofacial Pathology and Oral Microbiology Meenakshi Ammal Dental College & Hospital, MAHER Chennai Tamil Nadu India; ^6^ Centre for Biotechnology Anna University Chennai Tamil Nadu India; ^7^ Department of Biomedical Sciences, College of Medicine King Faisal University Al‐Ahsa Saudi Arabia; ^8^ ICMR‐Regional Medical Research Center Bhubaneswar Odisha India

**Keywords:** autophagy, cancer, celastrol, kaempferol, naringenin, piperine, resveratrol

## Abstract

**Background:**

Phytochemicals possess a wide range of anti‐tumor properties, including the modulation of autophagy and regulation of programmed cell death. Autophagy is a critical process in cellular homeostasis and its dysregulation is associated with several pathological conditions, such as cancer, neurodegenerative diseases, and diabetes. In cancer, autophagy plays a dual role by either promoting tumor growth or suppressing it, depending on the cellular context. During autophagy, autophagosomes engulf cytoplasmic components such as proteins and organelles. LC3‐II (microtubule‐associated protein 1 light chain 3‐II) is an established marker of autophagosome formation, making it central to autophagy monitoring in mammals.

**Objective:**

To explore the regulatory role of phytochemicals in LC3‐mediated autophagy and their potential therapeutic impact on cancer. The review emphasizes the involvement of autophagy in tumor promotion and suppression, particularly focusing on autophagy‐related signaling pathways like oxidative stress through the NRF2 pathway, and its implications for genomic stability in cancer development.

**Methods:**

The review focuses on a comprehensive analysis of bioactive compounds including Curcumin, Celastrol, Resveratrol, Kaempferol, Naringenin, Carvacrol, Farnesol, and Piperine. Literature on these compounds was examined to assess their influence on autophagy, LC3 expression, and tumor‐related signaling pathways. A systematic literature search was conducted across databases including PubMed, Scopus, and Web of Science from inception to 2023. Studies were selected from prominent databases, focusing on their roles in cancer diagnosis and therapeutic interventions, particularly in relation to LC3‐mediated mechanisms.

**Results:**

Phytochemicals have been shown to modulate autophagy through the regulation of LC3‐II levels and autophagic flux in cancer cells. The interaction between autophagy and other cellular pathways such as oxidative stress, inflammation, and epigenetic modulation highlights the complex role of autophagy in tumor biology. For instance, Curcumin and Resveratrol have been reported to either induce or inhibit autophagy depending on cancer type, influencing tumor progression and therapeutic responses.

**Conclusion:**

Targeting autophagy through LC3 modulation presents a promising strategy for cancer therapy. The dual role of autophagy in tumor suppression and promotion, however, necessitates careful consideration of the context in which autophagy is induced or inhibited. Future research should aim to delineate these context‐specific roles and explore how phytochemicals can be optimized for therapeutic efficacy. Novel therapeutic strategies should focus on the use of bioactive compounds to fine‐tune autophagy, thereby maximizing tumor suppression and inducing programmed cell death in cancer cells.

## Graphical abstract

Highlighting the promise of phytochemicals in modulating autophagy to combat cancer, the review explores their mechanisms of action and efficacy in preclinical studies. However, it also addresses the challenges inherent in translating these findings into clinical applications, including issues related to bioavailability, specificity, and potential adverse effects. Through a comprehensive analysis, this article underscores the potential of phytochemicals as valuable additions to cancer therapy while recognizing the hurdles that must be overcome for their successful implementation.

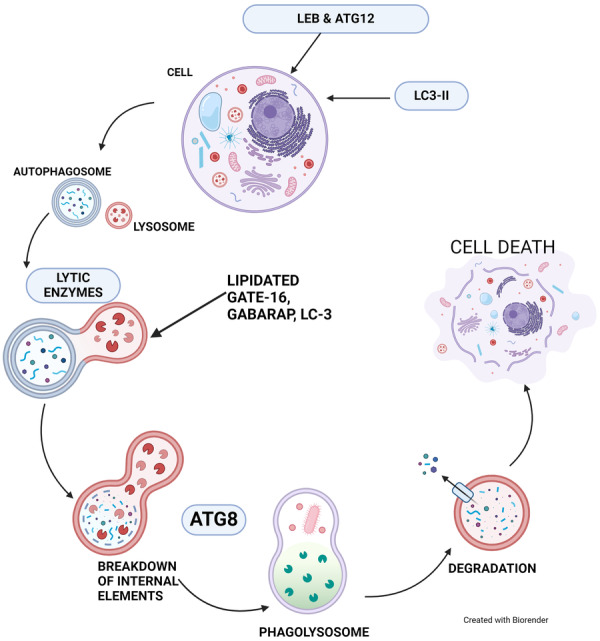

## 1 INTRODUCTION

To maintain metabolic balance and homeostasis, autophagy captures and degrades intracellular components, including proteins and organelles. As a result, autophagy plays an important role in protein and organelle quality control, by preventing gradual accumulation of damaged proteins and organelles in tissues *(1‐3)*. In yeast, more than 20 genes have been identified as autophagy‐related genes.^1^, ^2^, ^3^ Numerous yeast homologs of ATG genes identified and characterized in mammals. There's about 30% amino acid homology between microtubule‐associated protein 1 light chain 3 (LC3) and yeast Atg8. There are two forms of LC3‐I, LC3‐II.^4^, ^5^ Cells that contain LC3‐I found in the cytoplasm, whereas cells that contain LC3‐II are located within autophagosomes. Autophagy is stimulated by various stresses such as starvation, which converts LC3‐I to LC3‐II and upregulates LC3 expression.^6^ LC3 is a specific autophagosome marker.

Gly120, designated LC3‐I, is exposed at the C terminus of newly synthesized LC3 to a cysteine protease known as ATG4B. The LC3‐I ligand is conjugated to the head group of the lipid phosphatidylethanolamine by a series of ubiquitin‐like reactions involving the enzymes ATG7, ATG3 and ATG12‐ATG5‐ATG16.^7^ By lipidating the protein, LC3‐II is formed, which assists with its insertion into membranes. LC3 contains a LIR (LC3‐interacting region) domain at its N terminus which interacts with proteins containing this domain. Thus, LC3acts as an adaptor that facilitates the recruitment of cargo proteins with LIR motifs towards autophagosomes.^3^, ^8^ Some tumor tissues showed high levels of LC3 puncta and lipidated LC3 (LC3‐II), indicating the accumulation of autophagosomes, which prompted the first evidence that autophagy plays a role in the maintenance of established cancers.^9^, ^10^ Evidence suggests a link between diet and response to cancer treatment. Phytochemicals derived from dietary and medicinal plants were reported to inhibit the activity of a number of oncogenic molecules when consumed in sufficient amounts.^5^, ^11^, ^12^, ^13^, ^14^, ^15^, ^16^ The formation of the phagophore begins after being stimulated by the ULK1/2 complex. The enlargement of the phagophore facilitated by the complex of ATG12–ATG5‐ATG16L1, LC3‐II, ATG9, and the class III PtdIns3K complex. Ultimately, the enlarging membrane encloses its contents to create an autophagosome, and separation of LC3‐II from the outermost layer of this organelle. The external layer of the autophagosome will subsequently merge with the membrane of the lysosomal system, resulting in the creation of an autolysosome. Occasionally, the autophagosome may merge with an endosome, resulting in the formation of an amphisome, before merging with the lysosome. The autolysosome's contents are subsequently broken down and subsequently transported back into the cytoplasm for cellular reuse.^17^ In various human diseases, including cancer, the autophagy process is deregulated. Autophagy plays a dual role in cancer prevention and therapy, and either inhibiting or enhancing autophagic pathways can have therapeutic advantages.^18^ Recent evidence indicates that bioactive, natural product‐derived molecules, in addition to chloroquine (CQ) and hydroxychloroquine (HCQ), are involved in the regulation of autophagy in addition to these lysosomal inhibitors. By modulating several cellular signaling pathways and transcription factors, these molecules can modulate autophagic activity in vitro and in vivo.^19^, ^20^, ^21^, ^22^, ^23^, ^24^, ^25^

## 2 METHODOLOGY

### 2.1 Search methodology

When searching the electronic database, we used terms such as “LC3,” “Autophagy,” and “cancer therapy,” as well as “Phytochemicals,” “Kaempferol,” “Curcumin,” “Resveratrol,” and “Resveratrol” The study's relevance is assessed based on the title and abstract of the specific topic, which were collected until January 1, 2024. Two other researchers assessed the retrieved title and abstract to select the topic. If any instances of discrimination arise, they will be addressed and explained by the third author.

### 2.2 Inclusion criteria

We have chosen several studies according to the requirements for detailed examination. As an illustration, we have conducted research on the relationship between LC3‐autophagy and cancer therapy, as well as the use of phytochemicals to target LC3‐mediated autophagy for cancer treatment.

### 2.3 Exclusion criteria

We have excluded few of the studies which reported LC3‐autophagy and other disorders such as diabetes, neurodegenerative disease, and malignancies and its protection by phytochemicals.

## 3 LC3 STRUCTURE

**Figure 1:**
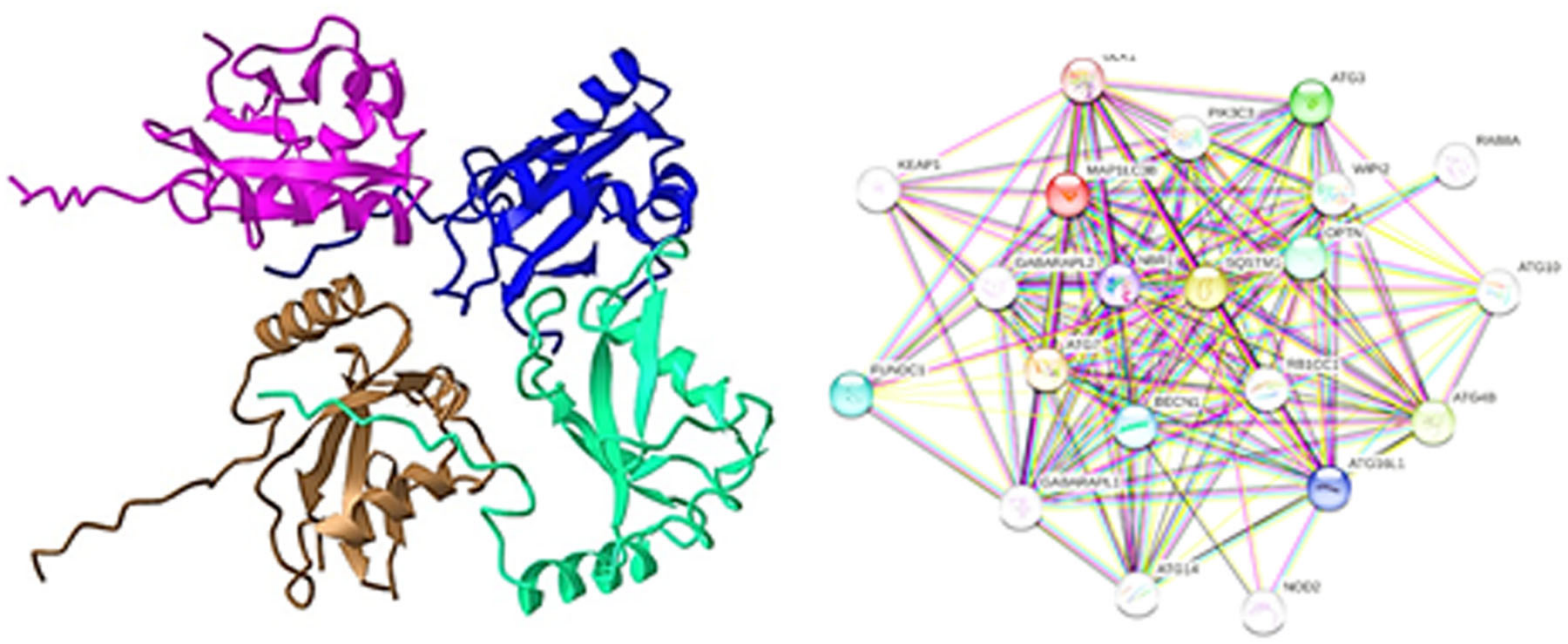
Structure of LC3 and protein−protein interaction network of the autophagy. LC3, light chain 3.

LC3 is a soluble protein with a molecular mass of 17 kDa that is widely distributed in mammalian tissues and cells in culture.^26^ In the early days of MAP1A and MAP1B purification, three light chains were identified as part of the purification process (LC1, LC2, and LC3).^27^ In the past, LC3 was thought to be involved in microtubule assembly and disassembly before its autophagy‐specific role as a mammalian Atg8 homologue was established.^28^ Atg7, an E1‐like enzyme, and Atg3, an E2‐like enzyme, catalyze two successive ubiquitylation‐like reactions to LC3‐II during autophagosomal membrane formation. Lysosomal proteases also degrade intra‐autophagosomal LC3‐II during the fusion of autophagosomes with lysosomes. In this sense, changes in cellular LC3‐II levels are linked to the dynamic turnover of LC3‐II via the lysosome, that is, autophagic activity^29^ (Figure 1).

## 4 MECHANISMS OF MAMMALIAN AUTOPHAGY MEDIATED BY LC3

**Figure 2:**
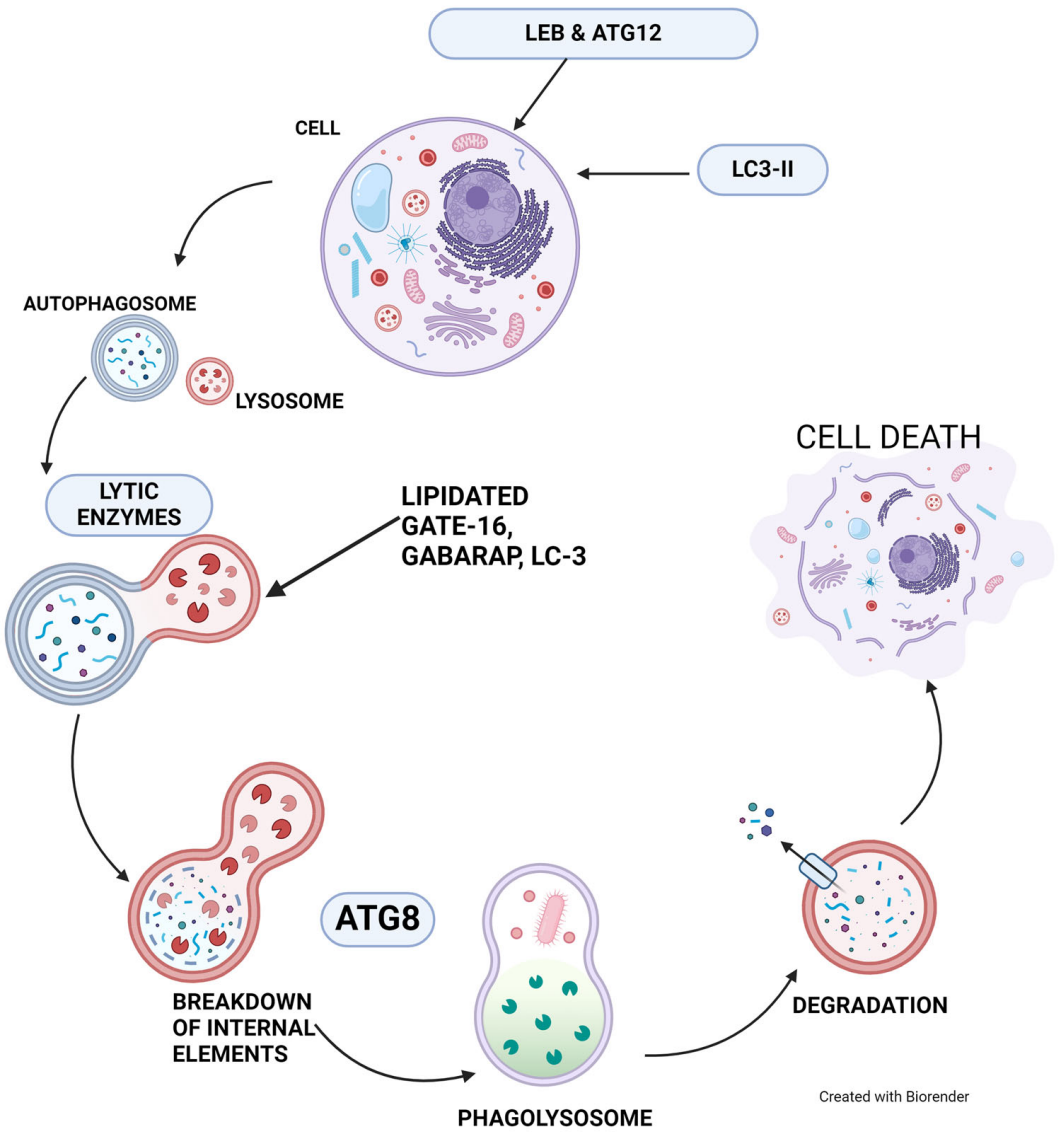
LC3 role in autophagosome formation and degradation. LC3, light chain 3.

Autophagy refers to the large‐scale breakdown of organelles and proteins which is a crucial process for maintaining cellular health, ensuring cell survival, promoting differentiation of cells, and facilitating growth in mammals.^30^ Autophagy is a process where a cup‐like structure, pre‐autophagosome engulfs various components in the cytoplasm, such as organelles.^3^ It then closes to form an autophagosome, which later merges with a lysosome. This fusion results in the breakdown of the internal elements of the autophagosome through the action of lytic enzymes in lysosomes. Two ubiquitylation‐like adjustments, LC3‐modification and Atg12‐conjugation, are necessary for the development of mammalian autophagosomes. LC3 is a protein that is similar to yeast Atg8 and is involved in the formation of autophagosomes. LC3‐II, a lipid‐modified variant of LC3, identified as a reliable indicator of autophagosomes in mammals. GATE‐16 and GABARAP, which are other homologues of Atg8, undergo modification by the similar method. The pattern of distribution of LC3‐II in rats that are not deprived is distinct from the distribution of the lipidated versions of GATE‐16 and GABARAP, specifically GABARAP‐II and GATE‐16‐II. This indicates that there is a functionality difference among these three changed proteins. The process of removing lipids from GABARAP‐II and LC3‐II is facilitated by hAtg4B.^3^, ^31^ It is important to mention that the levels of LC3, GABARAP, and GATE‐16 in cells indicate the amount of autophagic vacuoles (which include autophagosomes and autolysosomes), rather than the activity of autophagic proteolysis. The cellular concentrations of autophagosomal Atg8 homologues increase as a result of starvation‐induced production of autophagosomes. However, the levels of these homology decrease when autophagosomes mature into autolysosomes and the parts of the autophagosomes are degraded. One study demonstrates that cultured hepatocytes have a drop in LC3‐II level during long‐term fasting (greater than 4 h). However, when lysosomal proteins that inhibit proteinase are present, there is an accumulation of more LC3‐II.^3^, ^32^ This aligns with the earlier finding that the quantity of autophagic vacuoles significantly rises when the process of lysosomal proteolysis is hindered. This demonstrates that the molecular process of LC3‐modification, the relationship between mammalian Atg12‐conjugation, LC3‐modification, and LC3‐lipidation cycle, delipidation controlled by hAtg4B, LC‐3 autophagosome and its degradation of autophagy via LC‐3^3^ (Figure 2). Autophagy has studied extensively in various contexts such as neurological and neuromuscular conditions, cancer, as well as infections caused by viruses and bacteria.^3^

## 5 AN OVERVIEW OF AUTOPHAGY'S ROLE IN TUMOR PROMOTION

A number of studies have reported that autophagy can suppress early tumorigenesis,^33^ but it has also showed that autophagy can promote tumor growth across a bunch of different types of cancer,^33^, ^34^ as well as make it resistant to drugs. It might be partially due to inflammation, reactive oxygen species (ROS), and DNA damage promote tumorigenesis at early stages, but may be detrimental at later stages if autophagy has a dual role.^35^ Under stress, such as hypoxia and nutrient deprivation, autophagy showed to remove unfolded proteins and generates adenosine triphosphate by rapidly degrading endogenous substrates.^36^ It follows that autophagy is normally elevated in hypoxic regions of tumors, thereby promoting survival of cells.^37^ Many types of cancer are activated by autophagy, including those driven by K‐Ras protein (KRAS) and v‐Raf murine sarcoma viral oncogene homolog B1.^38^, ^39^ The autophagy pathway is important for the transformation of RAS. The effects of autophagy on KRAS‐induced tumorigenesis were not adequate to induce tumor transformation. A significant proportion of human PDAC tumors contain activating mutations of KRAS.^40^ This will result in increased autophagy. It was found that the inhibition of autophagy decreased tumor growth in vivo as well as growth of colonies in vitro.^41^, ^42^ A recent study concluded that autophagy is dispensable in KRAS‐mutant tumor cells, despite the fact that autophagy is necessary for tumorigenic growth. In addition, there is a question whether autophagy inhibition may be responsible for CQ and HCQ, its derivative with antitumor effects.^43^ A gene editing process that deletes autophagy genes may be prone to selective pressures that result in the resistant clones production.^43^ An intact immune system and homotypic tumor‐stromal interactions would not necessitate autophagy for formation of tumor.^44^, ^45^ It is likely that HCQ's antitumor properties are due to its ability to inhibit autophagy. It is also likely that CQ inhibits autophagy.^46^ Recent studies have revealed that autophagy‐related communication shows great potential as a therapeutic approach for treating hepatocellular carcinoma (HCC). Recent studies have emphasized the crucial importance of natural products that regulate autophagy in preventing the advancement of HCC. Natural compounds demonstrate lethal effects by inducing excessive autophagy, while also inhibiting autophagy to impede HCC cell proliferation, ultimately depriving HCC cells of vital energy. These effects have been linked to many signaling pathways, including as mitogen‐activated protein kinases (MAPK), phosphoinositide 3‐kinase/protein kinase B (PI3K/AKT), AMP‐activated protein kinase (AMPK), Beclin‐1, ferroautophagy, and Wnt/β‐catenin.^47^

## 6 THE ROLE OF AUTOPHAGY IN TUMOR SUPPRESSION

An autophagy possesses an impact of safeguarding against cancer. The initial evidence supporting the significance of autophagy in suppressing tumors was derived from investigations on the Beclin‐1 (BECN1) the gene responsible for producing beclin‐1. Examination of primary mammary tumor samples and breast cancer cell lines showed a high occurrence of eliminating one copy of the BECN1 gene. Additionally, it was observed that mice with just one copy of the BECN1 gene are more susceptible to developing tumors.^48^, ^49^, ^50^, ^51^ The deletion of one allele of the BECN1 gene may occur due to its relationship to the BRCA1 tumor suppressor gene on chromosome 17q21.^52^ The effects of disrupting autophagy upon the development of tumors vary depending on the autophagy genes and particular tissues involved. Preliminary investigations on the Becn1 gene in mice revealed, that one functional copy of Becn1 gene throughout the entire body led to the development of tumors in the liver, lymphatic tissue and lung. However, no tumors were observed in any other tissues or organs.^50^, ^53^ Furthermore, the only ablation of Atg7, lacking any other genetic alterations, specifically resulted in the development of malignancies exclusively in the liver.^54^ Numerous investigations have demonstrated that tumor‐suppressive mechanisms may control autophagy. Specifically, the prominent tumor‐suppressive gene transcription factor p53 recently reported to regulate autophagy through many mechanisms. Multiple studies have demonstrated the ability of tumor‐suppressive mechanisms to control autophagy. The prominent tumor‐suppressive transcription factor p53 showed to regulate autophagy through many mechanisms. Under normal conditions, cytoplasmic p53 inhibits autophagy.^55^ However, when triggered by cellular stress like DNA damage, the levels of p53 increase, leading to the stimulation of many genes that promote autophagy, such as PRKAB1 and DRAM1 genes.^56^, ^57^ The link connecting autophagy and p53 is mutually influential, as research indicates that ATG7 suppresses the activation of p53, whereas chaperone‐mediated autophagy leads to the breakdown of mutant p53.^58^, ^59^ Other investigations reveal selectivity for cells retaining deactivation of certain autophagy components through disease advancement, thus confirming the hypothesis of autophagy processes as effective tumor suppressor genes. The aforementioned investigations on allelic elimination of BECN1 in ovarian and breast malignancies serve as an illustration of this phenomenon. While a conclusive connection between tumor suppression and autophagy in human cancer has not been established, subsequent research has indicated the occurrence of allelic loss or reduced expression of BECN1 in various other forms of cancer.^60^, ^61^ Furthermore, new discoveries indicate that other autophagy genes, or factors that control ATG proteins, undergo mutations or become deactivated to avoid the tumor‐inhibiting effects of autophagy during the progression of tumor formation. Frame‐shift mutations was identified in multiple ATG genes, including ATG5, ATG2B, ATG12, and ATG9B in cases of liver and gastrointestinal malignancies. Additionally, downregulation of ATG7 identified in melanoma.^62^, ^63^ Furthermore, research conducted in mice revealed that the removal of mitophagy receptors Bcl‐2/adenovirus E1B 19 kDa protein‐interacting protein 3‐like or BNIP3 (also referred to as NIP3‐like protein X) in the presence of normal autophagy facilitated the growth of pancreatic cancer and breast cancer.^64^, ^65^

**Figure 3:**
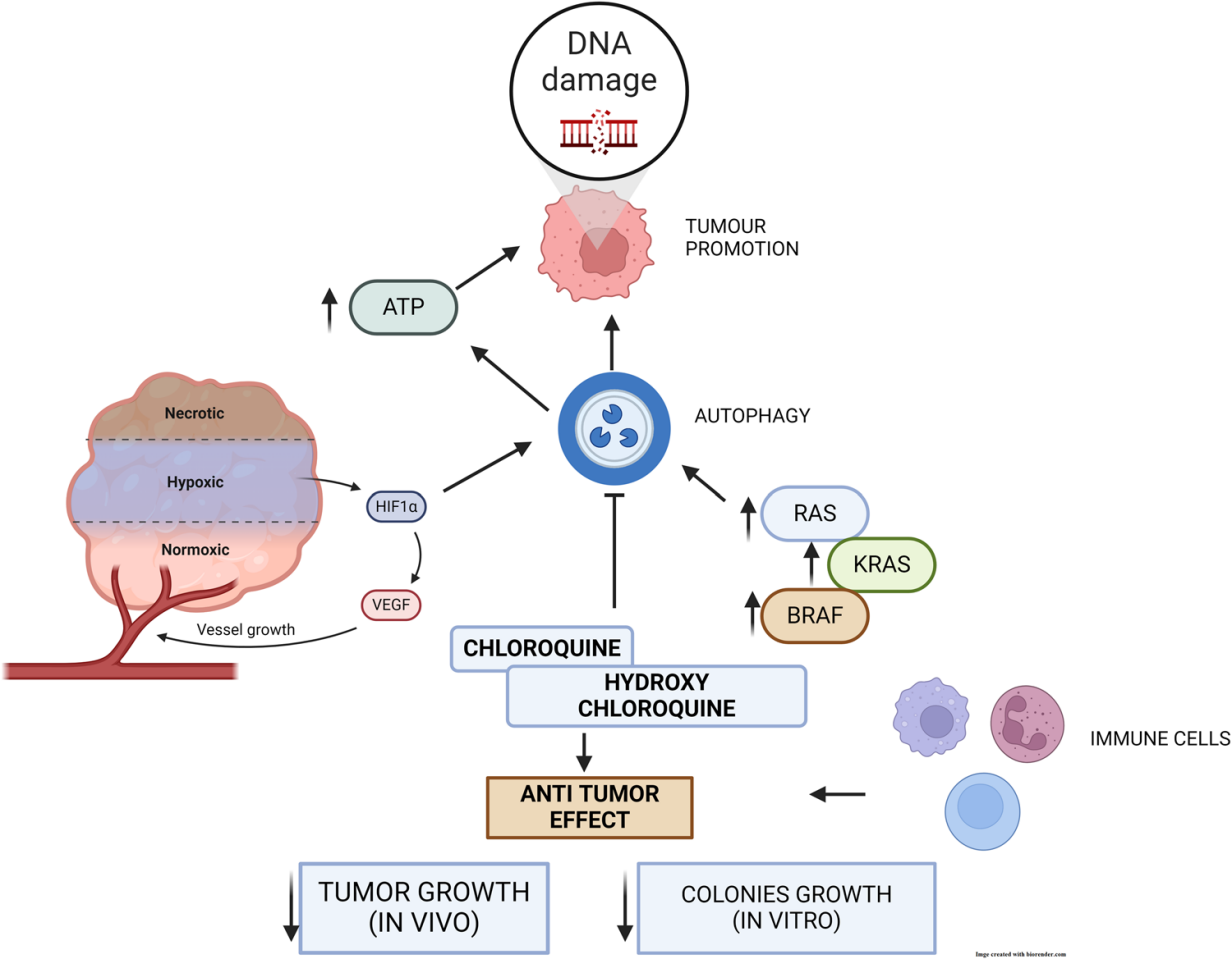
Role in autophagy in tumor formation.

A detailed discussion of the different kinds of autophagy, that are selective can be found further.^66^, ^67^, ^68^, ^69^ Two of these kinds are especially important for tumor suppression because they help to reduce cellular stress from ROS that could harm DNA and cause mutations and tumors to grow. One of these earliest and most well studied types of selective autophagy is mitophagy, which involves the intentional mitochondria removal. To maintain mitochondrial integrity, autophagic destruction of impaired mitochondria and replenishment by de novo synthesis constitute the main systems, since the cell's nucleus and cytoplasmic have more sophisticated and effective systems for repairing proteins and DNA.^70^ Accumulating impaired mitochondria found within the cells with critical autophagy enzymes removed, resulting in build‐up of ROS production and damage to DNA, provides support for the role of mitophagy as in suppression of tumors^71^, ^72^ (Figure 3).

## 7 IDENTIFYING AND TARGETING AUTOPHAGY‐REGULATING SIGNALING PATHWAYS

### 7.1 Mechanisms implicated in the suppression of tumor growth by autophagy

#### 7.1.1 Oxidative stress via NF‐E2‐related factor 2 (Nrf2) pathway and instability of genomes

An essential link connecting autophagy and tumor suppression is established through the control of ROS. Elevated levels of ROS enhance the pace of genetic mutations, leading to the heightened activation of oncogenes and thus promoting the development of cancer.^73^, ^74^ Mitochondria are recognized as the main producers of internal ROS, and their generation escalates with the aging or impairment of these compartments.^75^ Within this particular framework, autophagy prevents harm by selectively breaking down faulty mitochondria, a phenomenon referred to as mitophagy. Therefore, the suppression of autophagy promotes the occurrence of instability in genomic DNA by stimulating the induction of cancer‐causing genes.^76^, ^77^ Additionally, the harmful effects found in cells with impaired autophagy appear to rely on the development of ROS.^78^

Hence, the targeted elimination of possibly impaired mitochondria (mitophagy) decreases the excessive generation of ROS and consequently restricts the promoting effects that rely on the creation of these substances.^79^ Consequently, when autophagy suppressed in several models, there is an increase in more faulty mitochondria.^39^, ^42^, ^54^, ^80^, ^81^, ^82^

In addition, autophagy allows the breakdown of aggregates of protein. Malfunctions in the process of autophagy associated with the buildup of aggregates of protein and p62/SQSTM1 (autophagy substrate). These events linked to the endoplasmic reticulum (ER) stress, elevated formation of ROS, and the initiation of damaged reaction of DNA.^78^ The p62 gene is an exclusive target of autophagy, which is produced whenever autophagy is diminished. This structural protein contains a PB1 domain facilitating protein oligomerization, along with a UBA domain crucial for binding up the LIR and polyubiquitinated proteins necessary for LC3 interaction.

Due to these factors, p62 promotes the targeted breakdown of both proteins (polyubiquitinated) and organelles, such as mitochondria.^83^, ^84^ Notably, levels of p62 frequently increased in human malignancies. Furthermore, the growth of tumors in cells without autophagy is counteracted by the genetic deactivation of p62 in different experimental setups. This indicates that the buildup of p62 contributes to the development of tumors in this particular scenario.^39^, ^54^, ^78^, ^85^ Furthermore, the increase of p62 enhances the stability and transcriptional activation through NRF‐2 by interacting to Keap‐1, which is the primary inhibitor of NRF‐2. By upregulating antioxidants defense proteins, the growth of tumors might be initiated.^85^, ^86^, ^87^ More precisely, the excessive production of p62 and the stimulation of NRF‐2 are essential for the observed proliferation of cells with HCC in the absence of anchoring.^85^

#### 7.1.2 A link between autophagy, inflammation, and necrosis

It is important to know, that cytokines and inflammatory cells play a significant role in tumor formation. This is due to a circumstance that fosters inflammation encourages the development and persistence of cancerous cells, stimulates the formation of new blood vessels, facilitates the spread of cancer to other parts of the body, and alters the effectiveness of drugs.^88^ Autophagy suppression in apoptosis‐deficient malignancies is being demonstrated to result in local inflammation, growth of the tumor and necrotic death of cells, development in several models.^37^ The findings indicate that autophagy might play a role in inhibiting tumor development by limiting local inflammation and tumor necrosis.^89^ The connection between the anti‐inflammatory properties impact of autophagy and the elimination of dead cells^90^ have proposed based on research involving embryonic stem cells (Atg5 −/−). Results revealed impairments in the elimination of apoptotic corpses throughout embryonic growth.^91^ Furthermore, a complex correlation between autophagy and other facets of the immune system's defense is being observed, potentially enhancing the tumor‐suppressive function of autophagy.^92^

### 7.2 Autophagy's involvement in tumor growth is mediated by immune response

Certain phagocytic vesicles are adorned with LC3 has revealed an unconventional function of ATG molecules that extend over the creation of autophagosomes.^93^ Further studies extensively elucidated the mechanism of LC3‐associated phagocytosis (LAP) and also discovered similar LC3 adhesion on the endosomes^94^ named LC3‐linked endocytosis (LANDO),^95^ as well as a process called LDELS.^96^ These processes involve the attachment of ATG8 proteins onto individual membranes, which recently termed conjugation of ATG8 to single membranes (CASM).^97^ CASM procedures can be differentiated based on the necessity of particular ATG complexes.^98^ LAP promotes the recruitment of lysosomes to phagosomes and the breakdown of their contents, therefore reducing proinflammatory signals by aiding in the removal of phagocytosed substances. Deletion of rubiconautophagy regulator in myeloid cells was demonstrated to inhibit LAP, leading to an enhancement of type I interferon signaling in macrophages associated with tumors. This, in turn, resulted in the reduction of tumor development through T cell‐mediated mechanisms.^99^ Remarkably, there is a noticeable increase in the Rubicon expression, which is necessary for LAP and not for canonical autophagy,^99^, ^100^, ^101^ in several types of malignancies such as liver, breast and stomach. This enhanced expression is linked to a worse outcome in individuals.^102^ LC3‐associated activities may additionally offer non‐degradative functions. LANDO was reported to control the process of recycling receptors on the cell surface. Blocking LANDO in cells with myeloid cells hindered the reuse of receptors that are responsible for taking in of Aβ amyloid, which is related to the development of Alzheimer's disease. These receptors include toll like receptor 4, triggering receptor expressed on myeloid cells 2 (TREM2) and CD36.^95^ Consequently, inhibiting LANDO leads to elevated amounts of Aβ amyloids outside of cells and triggers a response of inflammation in the brains of mice. Notably, it has been demonstrated that the presence of TREM2 is associated with an unfavorable cancer outcome.^103^

## 8 NATURAL PRODUCTS AS MODULATORS OF LC3 MEDIATED AUTOPHAGY

### 8.1 Kaempferol

Kaempferol is a member of the flavonoid group and is commonly utilized in conventional medical practices. The anticancer actions of kaempferol involves in inducing an arrest in cell cycle and promoting autophagy‐dependent cell death.^11^ Kaempferol showed to reduce the viability of cells and to triggere cell cycles arrest in the G2/M phase. Kaempferol participates in the process of autophagy. Observations were made of compartments at lysosomal, vacuoles (double‐membrane), cleavage of microtubule‐associated protein 1 LC3 and acidic vesicular organelles. Kaempferol reported to elevate the levels of LC3‐II, p‐AMPK, Atg7, Atg5, beclin1, and Atg12 proteins, while suppressing the protein levels of cyclin B, CDK1, p‐mammalian target of rapamycin (mTOR) and p‐AKT. The combined effects of the expression of CDK1/cyclin B and the signaling of AKT and AMPK pathways were responsible for the cell cycle arrest at G2/M phase and mediates cell death through autophagy produced by kaempferol in hepatic cancer cells of human (SK‐HEP‐1).^104^

**Figure 4:**
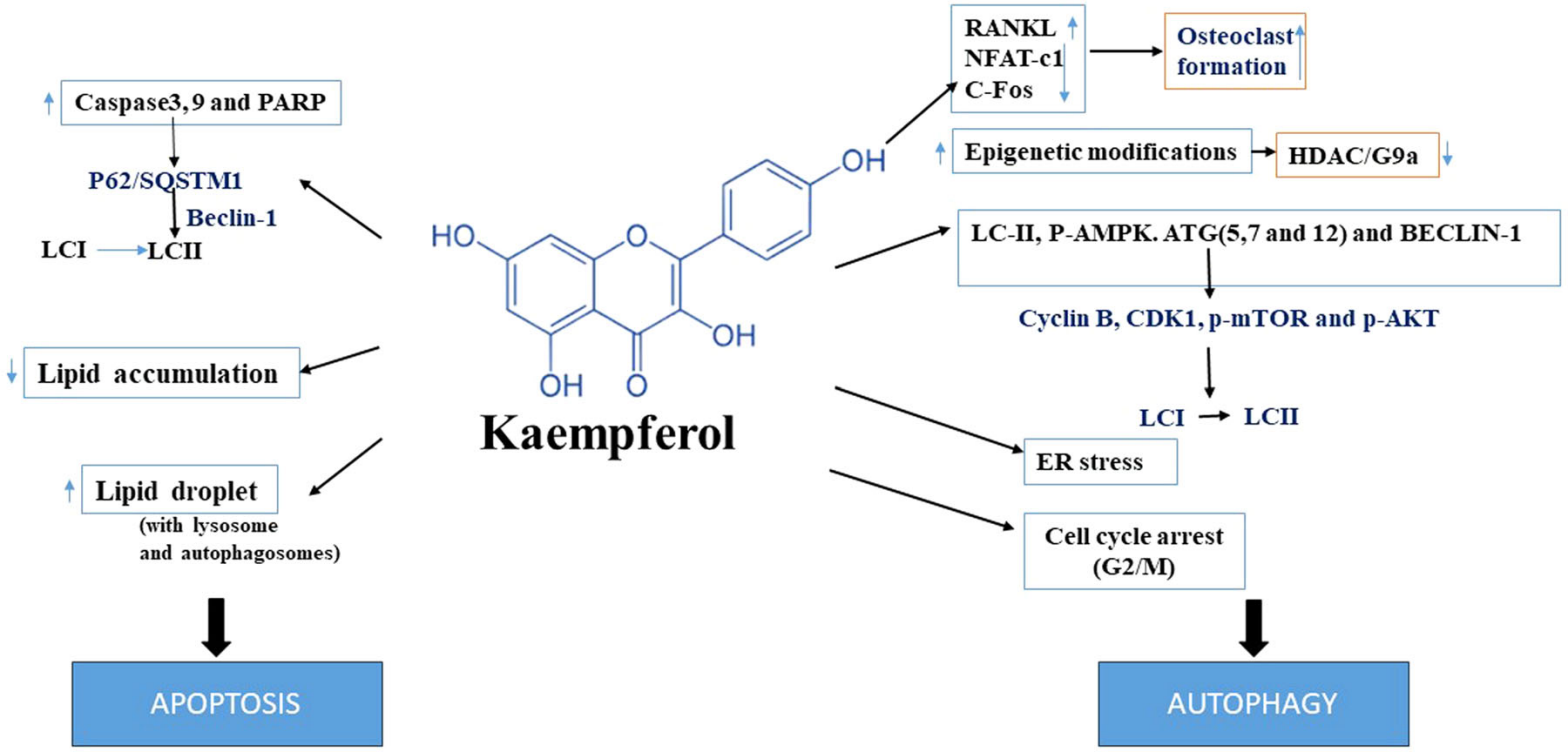
Kaempferol targeting on autophagy mechanism in cancer.

Kaempferol enhances the process of autophagy and induced cell death, leading to an upsurge in the conversion of LC3‐I to LC3‐II and a decrease in the p62 level of expression in gastric cancer (GC). Kaempferol triggers the autophagy‐dependent cell death by activating signaling such as IRE1‐JNK‐CHOP, which indicates a response to ER stress (Table 1). ER stress inhibition effectively reduced the autophagy generated by kaempferol, resulting in extended survival of cells and suggesting the occurrence of the autophagy‐dependent cell death. Additionally, the study showed that kaempferol could induce epigenetic modifications via inhibiting G9a, a component of the HDAC/G9a axis, and triggers the autophagic death of cells.^105^, ^131^ Taken together, kaempferol stimulates the signaling pathway via IRE1‐JNK‐CHOP, facilitating the transmission of signals through the cytosolic to the nuclei. Additionally, inhibiting G9a triggers autophagy‐dependent cell death in Gastic cancer cells.^105^ The presence of kaempferol inhibits the process of autophagy, which in turn decreases the formation of osteoclasts in RAW 264.7 cells. Kaempferol exerted a dose‐dependent effect on RAW 264.7 cells by decreasing the viability of cells, inhibiting osteoclasts, and inducing autophagy and apoptotic‐related components. Kaempferol showed to enhance the process of apoptosis and autophagy in the formation of osteoclasts. Kaempferol hindered the process of cell differentiation (RAW 264.7) into osteoclasts, which was triggered by RANKL, via suppressing the NFAT‐c1, c‐Fos and TRAF6. Kaempferol significantly suppressed the activity of phospho‐cFos and c‐Fos. Kaempferol suppressed the activity of p62/SQSTM1, beclin‐1 and converted LC3‐II from LC3‐I (Figure 4). These findings indicate that kaempferol inhibits the process of osteoclast development by degrading p62/SQSTM1. p62/SQSTM1, beclin‐1 and ATG5 are commonly blocked because 3‐Methyladenine (3‐MA) has the ability to impact lysosomes throughout the process of autophagy independently. The transformation of LC3‐I to LC3‐II was impeded by 3‐MA. Additionally, 3‐MA significantly decreases the levels of p62/SQSTM1 and c‐Fos, while simultaneously activating the apoptosis‐associated signaling pathway such as caspase‐3, poly (ADP‐ribose) polymerase (PARP), and caspase‐9. Thus, kaempferol suppressed autophagy and enhanced cell death via apoptosis, while also inhibiting osteoclastogenesis (RAW 264.7 cells).^132^ The administration of kaempferol resulted in reduction in the accumulation of lipids and a rise in the droplets of lipids with lysosomes and autophagosomes in β‐cells exposed to palmitic acid. Kaempferol inhibits abnormal buildup of lipids and ER stress, hence improving the function of β‐cells through the activation of AMPK‐lipophagy.^133^ Kaempferol promotes cell viability in none malignant cells by suppressing the C/EBP homologous protein (CHOP) and increasing the levels of GRP78. Conversely, in cancer cells, kaempferol induces cell death by activating the response of unfolded proteins and increasing the levels of CHOP.^134^

**Table 1:** Phytochemicals targeting LC3 mediated autophagy signaling in cancer therapy.

Name of compounds	Target gene	Cancer type	Model	References
Kaempferol				
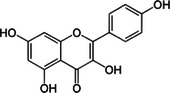	IRE1‐JNK‐CHOP	Gastric Cancer	In vitro (AGS and SNU‐638)	[105]
	AMPK	Liver cancer	In vitro (SK‐HEP‐1)	[106]
	CCAAT/enhancer‐binding protein homologous protein	Liver cancer	In vitro (HepG2, Huh7)	[107]
	PI3K/AKT/mTOR	Lung cancer	In vitro (A549)	[108]
	LC3/p62	Glioblastoma	In vitro (U251 and U87 MGs)	[109]
Resveratrol				
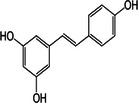	NAF‐1	Pancreatic cancer	In vitro (Panc‐1, MiaPaCa‐2, BxPC‐3, CF PAC‐1, and SW1990)	[110]
	SREBP1	Oral cancer	In vitro (Ca9‐22)	[111]
	PI3K‐AKT, JAK‐STAT	Ovarian cancer	In vitro (SKOV3 and OVCAR3)	[112]
	AMPK/mTOR	Lung cancer	In vitro (A549)	[113]
	Fas/Cav‐1	Lung cancer	In vitro (A549)	[114]
	LKB1‐AMPK‐mTOR	Leukemia	In vitro (HL‐60)	[115]
	LC3B	Colon cancer	In vitro (HCT116 and HT29)	[116]
	WIPI‐1/LC3	Osteosarcoma, Cervical and breast cancer	In vitro (U2OS, HeLa, G361 and MCF‐7)	[117]
	AMPK and mTOR	Multiple myeloma	In vitro U266, RPMI‐8226, and NCI‐H929	[118]
	AMPK and Akt/mTOR	Oral cancer	In vitro (CAL27)	[119]
Celastrol				
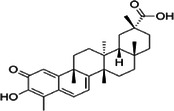	LC3B‐II	Osteosarcoma	In vitro (HOS and MG‐63)	[120]
	LC3, P62 and Beclin‐1	Glioblastoma	In vitro (U251)	[121]
	ATG5 and ATG7	Prostate cancer	In vitro (LNCap)	[122]
Curcumin				
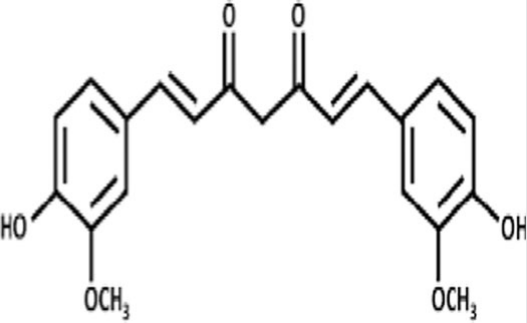	LC3‐II	Papillary thyroid carcinoma	In vitro (BCPAP)	[123]
	GD3	Lung cancer	In vitro (A549)	[124]
	LC3‐ II	Liver cancer	In vitro (HepG2)	[125]
	LC3‐ II	Oral cancer	In vitro (OSCC)	[126]
	LC3‐II	Colon cancer	In vitro (HCT116)	[127]
	p62, beclin‐1	Colon cancer	In vitro (HCT116 and HT29)	[128]
	JNK	osteosarcoma	In vitro (MG63)	[129]
	LC3‐II	Pancreatic cancer	(PANC1 and BxPC3)	[130]

Abbreviations: AMPK, AMP‐activated protein kinase; mTOR, mammalian (or mechanistic) target of rapamycin; PI3K/AKT, phosphoinositide 3‐kinase/Protein Kinase B.

### 8.2 Resveratrol

**Figure 5:**
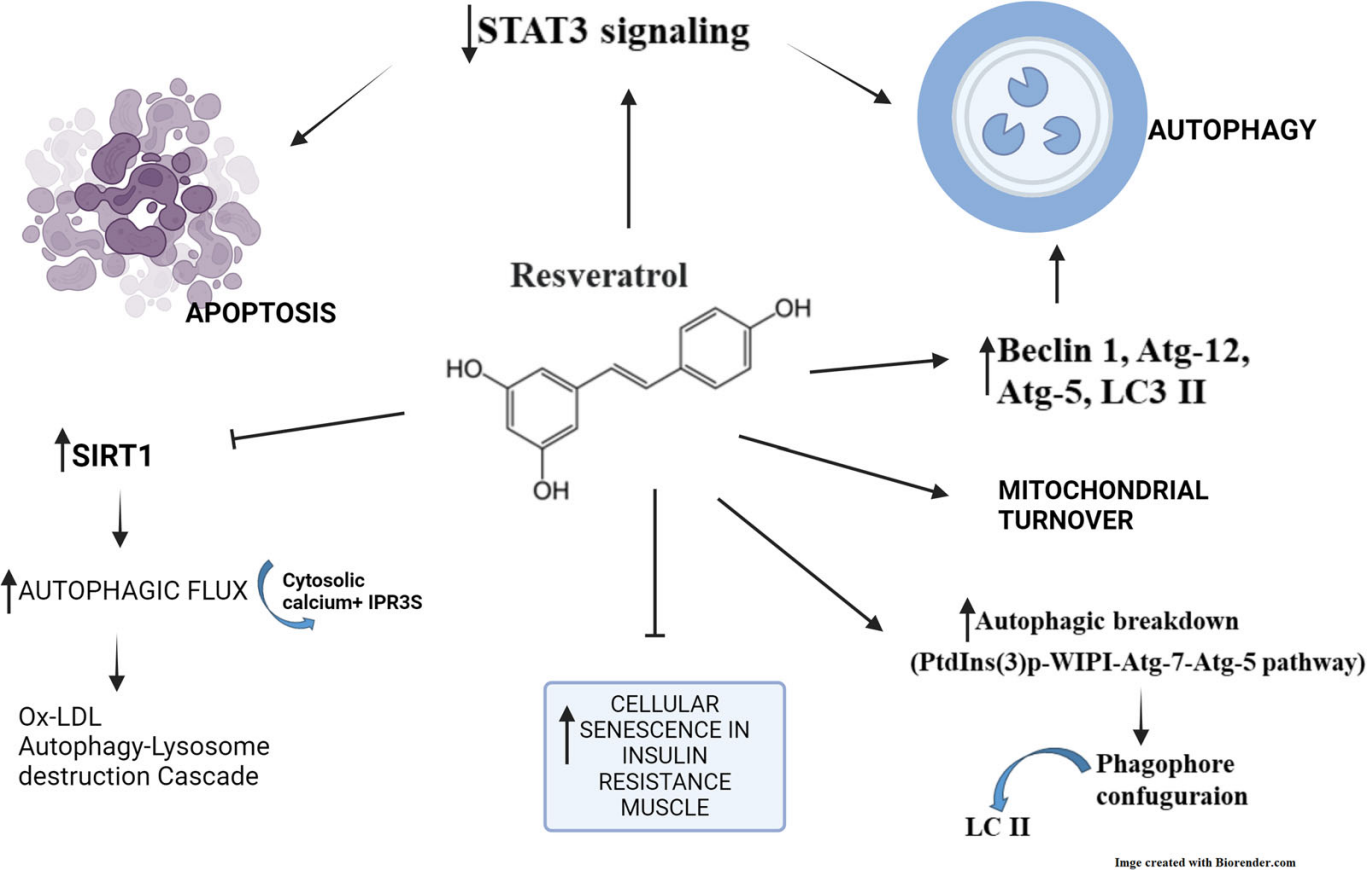
Resveratrol targeting on autophagy mechanism in cancer.

Resveratrol reported to enhance a specific type of autophagic breakdown that occurs after the PtdIns(3)P‐WIPI‐Atg7‐Atg5 pathway. This is achieved by interacting with a particular type of LC3‐II, which might be formed at membrane sources that are separate from the usual phagophore configurations.^117^ In addition, resveratrol enhanced the expression of mRNA genes, such as Beclin‐1, Atg12, Atg5, and LC3‐II involved in autophagy, in CAR cells. Resveratrol is expected to trigger autophagic and cell death by apoptosis in resistant to drugs in oral cancer cells.^119^ Resveratrol enhances apoptotic and autophagic processes while deactivating STAT3 signaling. Further, resveratrol induces the mitochondrial turnover, which can be detrimental to the survival of OC cells and increases their sensitivity to resveratrol.^135^ In HUVECs, resveratrol increased the level of SIRT1 expression, improved the function of the lysosome, improved the disrupted autophagic flux caused by Ox‐LDL, and facilitated the breakdown of Ox‐LDL via the autophagy‐lysosome destruction cascade.^136^ Resveratrol significantly enhances the rate at which autophagic flux occurs. Autophagy triggered by resveratrol is contingent upon cytosolic calcium ion signaling. Cytosolic Ca^2+^ and IP3Rs signaling play a crucial role in promoting autophagic transition, not merely when mTOR is inhibited but when noncanonical autophagy stimulants such as resveratrol are used.^137^ The inhibition of autophagic flux plays a critical role in the development of cellular senescence and resistance to insulin in muscle cells induced by palmitate (Figure 5). Resveratrol has the potential to effectively restore autophagic flow, which might represent a viable strategy for preventing cellular senescence and improving the resistance to insulin in muscle.^138^

### 8.3 Celastrol

Celastrol was demonstrated to exhibit strong regulatory actions in autophagy and has a wide range of potential pharmaceutical applications. Celastrol is a valuable tool for investigating autophagy.^139^ The induction of autophagy by celastrol significantly reduces the production of ROS and provided protection against damage to cells caused by the use of hydrogen peroxide. This effect was achieved by raising the ratio of LC3II to LC3I and enhancing the expression of Beclin1. These findings indicate that celastrol has potential for therapy in the treatment of macular degeneration caused by age through its ability to modulate autophagy.^140^ Celastrol caused a halt in the G2/M stage of the cell cycle and triggered programmed cell death. Celastrol enhanced the generation of autophagosomes, buildup of LC3B, and the production of p62 polypeptide. The reciprocal inhibition of apoptosis and autophagy was reported in cells of glioma treated with celastrol. In addition, celastrol triggered JNK stimulation and ROS generation. The autophagy and apoptosis induced by celastrol were greatly reduced by JNK as well as ROS inhibiting agents, although Akt as well as mTOR inhibiting agents produced contrasting impacts.^121^ Celastrol promotes the suppression of the tumors and triggers autophagy in cells with cancer via modulating signaling networks including Akt/mTOR, Beclin‐1, NF‐κB, ROS, HSP90, MAPK, and the proteasome. Based on the information provided, it can be concluded that celastrol possesses anticancer properties, affects different cellular pathways, acts as a modulator of autophagy in cancer treatment, and aids in reducing resistance to multiple drugs in cancer cells.^141^ The androgen receptor suppresses autophagy by activating miR‐101. Thus, combining miR‐101 with celastrol could be a potentially effective treatment for prostate tumors.^122^ Celastrol acts as a ligand for the nuclear target Nur77. The researchers have discovered a mechanism through which celastrol stimulates the passage of Nur77 to mitochondria from the nucleus. In this process, Nur77 is ubiquitinated by the protein TRAF2. Ubiquitinated Nur77 subsequently binds with p62/SQSTM1, resulting in the autophagy of defective mitochondria and the reduction of inflammation.^142^ Celastrol stimulated calcium signaling to promote the death of autophagic cells in RASFs/RAFLS and improved arthritis in AIA rats through the involvement of calcium‐dependent binding proteins. Thus, manipulating calcium signaling could be a promising approach for enhancing the effectiveness of antiarthritic medications.^143^

### 8.4 Curcumin

Curcumin, a polyphenol molecule (hydrophobic) derived from turmeric, exhibits many therapeutic actions. Curcumin effectively suppresses the proliferation of NSCLC A549 cells by triggering both autophagy and apoptosis via inhibiting the PI3K/Akt/mTOR cascade.^144^ The cell death caused by curcumin was similarly suppressed when rapamycin, an inducer of autophagy, was co‐incubated. When cells were cultured in a medium lacking serum, the amount of LC3‐II raised. The cell death reduced by suppressing curcumin‐autophagy by the use of siRNA targeting Atg5 or Beclin1. Thus, curcumin‐induced autophagy enhances the death of tumor cells, but the fundamental level of autophagy remains unaffected in serum‐deprived or rapamycin‐treated situations.^145^ Curcumin was shown to suppress the activity of autophagic indicators, specifically Beclin1 and LC3II/LC3I. Specifically, curcumin causes an increase in accumulation of p62 and its Connections between Nrf2 and Keap1 resulted in the translocation of Nrf2 to the nucleus. This translocation was preceded by an upsurge in the levels of heme oxygenase 1 and nicotinamide adenine dinucleotide phosphate hydrogen quinone dehydrogenase (Nqo1). Curcumin treatment resulted in an increase in the protein kinase B (p‐AKT) and phosphorylation of phosphatidylinositol 3 kinase (p‐PI3K). The 3‐MA, autophagy inhibitor stimulated the PI3K/AKT and keap‐1/Nrf2 signaling pathways.^146^ Curcumin demonstrates neuroprotective properties towards ischemia‐reperfusion by regulating the mutually reinforcing interaction between HIF‐1α and autophagy.^147^ The generation of ROS and the development of autophagosomes (via conversion of LC3‐I to LC3‐II), which occurs as a result of curcumin treatment, were significantly inhibited by N‐acetylcysteine, SKQ1, MitoQ10, BAPTA‐AM, EGTA‐AM, and ruthenium red, all of which are particular antioxidants or inhibitors targeting different cellular components. Autophagy produced by curcumin was ineffective in saving all of the cells, and the majority of cells experienced type II cell death after the early autophagic activities. This indicates that curcumin operates through a fail‐secure system controlled by autophagy, which suggests that curcumin has medicinal potential.^148^

### 8.5 Naringin and naringenin

Naringenin induces upregulation of LC3 protein and enhances the expression of Beclin‐1, p62, and ATG5 leading to notable efficacy in the treatment of osteosarcoma.^149^ Prior research has established that autophagy plays a crucial role as an indicator for the downstream of the pathway consisting of PI3K/AKT/mTOR in the process of drug‐induced death of cancer cells. By blocking the PI3K/AKT signal, it triggers autophagy and effectively hinders the proliferation of GC cells. The stimulation of the PI3K‐Akt‐mTOR cascade and the suppression of autophagy are responsible for the protective properties of naringenin and naringin. Moreover, research has shown that naringin influences the process of autophagosome development. Prior studies demonstrated that naringin triggers autophagy by upregulating the expression of Beclin‐1 protein, which in turn leads to the conversion of cytoplasmic LC3‐I protein into the autophagic variant LC3‐II. Thus, Naringin and Naringenin induce apoptosis in cancer cells by suppressing autophagy via pathways of signaling, so greatly influencing the efficacy of tumor therapy, particularly when used in together with other chemical agents.^150^

### 8.6 Carvacrol

Carvacrol was found to possess antitoxic and anti‐inflammatory properties. It was observed to decrease the oxidative harm in PC12 cells caused by cadmium through caspase‐dependent and apoptosis‐independent routes. Exposure to carvacrol resulted in an increase in the expressions of glutathione reductase and the cellular level of glutathione. Additionally, carvacrol improved the magnitude of DNA fragmentation produced by cadmium. Carvacrol enhanced the suppression of nuclear factor kappa‐light‐chain‐enhancer of activated B cells (NFKB), the reduction of protein kinase B (Akt), mTOR, and extracellular signal‐regulated kinase‐1. Carvacrol inhibited the activation of caspase 3, decreased the amounts of apoptosis‐inducing factor and cytochrome c in the cytoplasm, and elevated the amount of metallothionein inside the cells. The study investigates the ability of carvacrol to protect toward resistance to cisplatin in HeLa cells by promoting autophagy through the ERK1/2 pathway.^151^

### 8.7 Farnesol

Farnesol, an isoprenoid alcohol, effectively triggers apoptosis and cell cycle arrest in various types of carcinoma cells. Furthermore, studies have shown that farnesol can effectively impede the development of tumors in multiple animal models, indicating its potential as an antitumour and chemopreventive treatment in living organisms. The inhibition of growth and apoptosis‐inducing properties of farnesol are linked to many cellular and biochemical processes. Farnesol stimulates the triggering of the NF‐κB signaling cascade and several genes that are targeted by NF‐κB. The MEK1/2‐MSK1 signaling pathway was found to be necessary for the phosphorylation of p65/RelA to achieve optimal activation of NF‐κB. The occurrence of farnesol‐mediated apoptosis was observed to be associated with the enlargement of the apoptosome in certain cells. This text discusses the biochemical and cellular processes that are controlled by farnesol, specifically in regard to its ability to restrict growth, promote apoptosis, and have antitumor effects.^152^ Farnesol with respect to autophagy and anticancer mechanism needs to be elucidated.

### 8.8 Piperine

Piperine suppresses the growth and multiplication of prostate cancer cells by triggering autophagy, as evidenced by the rise in LC3II levels in PC3 and LNCaP cells. This may be verified by co‐administeration of piperine with the lysosomal blocker CQ, which results in a further increase in the production of LC3B puncta at the cellular level in LNCaP and PC3 cells. This suggests that piperine stimulates autophagy flux. Piperine is a potent inhibitor of mTOR, which is a crucial suppressor of autophagy. Piperine demonstrates inhibitory activity on mTORC1 in HT‐29 and Caco‐2 cells. Piperine inhibits the proliferation of HT‐29 cancer cells by promoting autophagy and activating proapoptotic elements of ER stress, including GRP78, CHOP, JNK and IRE1α. In addition, it suppresses the phosphorylation of Akt and the production of survivin. CHOP, often referred to as GADD,^129^ is a transcription factor belonging to the C/EBP family. It has a crucial function in apoptosis triggered by ER stress.^153^ Piperine has been demonstrated to stimulate autophagy in cells with cancer by suppressing mTORC kinase expression, hence facilitating the creation of autophagosomes. Piperine, in this situation, suppressed the activity of thioredoxin reductase, raised levels of ROS, decreased the potential of the mitochondrial membrane, and triggered autophagy in Bel‐7402/5‐FU cells by controlling autophagy‐related proteins beclin‐1, p62, and LC3. Experimental studies conducted in living organisms have shown that piperine triggers the process of autophagy by blocking the signaling of PI3K, resulting in a reduction in the growth of oral cancer tumors.^154^

## 9 CONCLUSION

Autophagy is a cellular process that breaks down and removes defective components within the cell, such as cytoplasmic sections, organelles, and proteins that have misfolded. This is achieved through the fusion of double‐membrane vesicles called autophagosomes, which contain the targeted cargo, with lysosomes. The purpose of autophagy is to preserve cellular homeostasis. This review article specifically mentioned about the few points such as autophagy is triggered by various stresses, such as starvation, which leads to the conversion of LC3‐I to LC3‐II and an increase in LC3 expression. Changes in cellular levels of LC3‐II are associated with the dynamic turnover of LC3‐II through the lysosome, which is the process of autophagic activity. The molecular process of LC3‐modification, the relationship between mammalian Atg12‐conjugation, LC3‐modification, and the LC3‐lipidation cycle, as well as the dilapidation controlled by hAtg4B, LC‐3 autophagosome, and its degradation of autophagy via LC‐3 are all interconnected. A recent study concluded that autophagy is not essential in KRAS‐mutant tumor cells, despite its necessity for tumorigenic growth. Additionally, there is uncertainty on whether the inhibition of autophagy could be the cause of the anticancer effects of CQ and HCQ, which is a derivative of CQ. The presence of dysfunctional mitochondria in cells lacking essential autophagy enzymes leads to the accumulation of ROS and DNA damage. This supports the idea that mitophagy plays a role in suppressing tumors. The elevation of p62 levels enhances the stability and transcriptional activation of NRF‐2 by interacting with Keap‐1, which is the main inhibitor of NRF‐2. By increasing the production of proteins that protect against oxidative damage, the development of tumors may be triggered. There is a complicated relationship between autophagy and other aspects of the immune system's defense, which could potentially enhance the tumor‐suppressing role of autophagy. They selected few natural compounds and their mechanisms of action as modulators of autophagy. Curcumin, Celastrol, Resveratrol, Kaempferol, Naringenin, carvacrol, Farnesol and Piperine are examples of compounds that operate through distinct routes via LC3 conversion mechanism in cancer therapy.

## FUTURE RESEARCH

This study focuses on the LC3 family and current progress in comprehending their functions in the process of autophagy, particularly in relation to phytochemicals targeting. But still there are some lacking in this studies such as the precise functions of the GABARAP, GATE‐16, and Atg8 homologues in the process of autophagy are not yet well comprehended. Initially, these proteins were discovered in the nervous system, just like LC3. It was found that soluble GATE‐16 and GABARAP have separate functions in plasma membrane receptor and membrane trafficking activity, respectively. It is necessary to clearly define the relationship between phytochemicals and the proteins LC3, GABARAP, and GATE‐16. It is crucial to mention that the present research did not define the levels at which autophagy is triggered or suppressed by natural substances. Conducting research in this field is crucial to gain understanding of the role underlying with LC3‐ mediated autophagy with respect to the phytochemicals. Once this mechanism is identified, we can utilize it in designing therapeutic target for LC3 mediated autophagy in different diseased condition especially for the cancer patients in future.

## AUTHOR CONTRIBUTIONS

*Conceptualization*: Peramaiyan Rajendran, Basem M. Abdallah, and Kaviyarasi Renu. *Methodology*: Peramaiyan Rajendran, Enas M. Ali, and Basem M. Abdallah. *Validation*: Marwa Azmy M. Genena, Enas M. Ali, and Parthasarathi Barik. *Formal analysis*: Sujatha Tejavat, Vishnupriya Veeraraghavan, and Parthasarathi Barik. *Writing—original draft preparation*: Peramaiyan Rajendran, Ashok Kumar Sekar, and Kaviyarasi Renu. *Writing—review and editing*: Peramaiyan Rajendran and Basem M. Abdallah. *Supervision*: Peramaiyan Rajendran. *Project administration*: Peramaiyan Rajendran. *Funding acquisition*: Peramaiyan Rajendran. All authors have read and agreed to the published version of the manuscript.

## CONFLICT OF INTEREST STATEMENT

The authors declare no conflict of interest.

## Data Availability

Data will be made available on request.
